# *Rickettsia conorii* Indian Tick Typhus Strain and *R. slovaca* in Humans, Sicily

**DOI:** 10.3201/eid1806.110966

**Published:** 2012-06

**Authors:** Alessandra Torina, Isabel G. Fernández de Mera, Angelina Alongi, Atilio J. Mangold, Valeria Blanda, Francesco Scarlata, Vincenzo Di Marco, José de la Fuente

**Affiliations:** Intituto Zooprofilattico Sperimentale della Sicilia, Palermo, Sicily, Italy (A. Torina, A. Alongi, V. Blanda, V. Di Marco);; University of Palermo, Palermo (V. Blanda);; Instituto de Investigación en Recursos Cinegéticos (IREC-CSIC-UCLM-JCCM); Ciudad Real, Spain (I.G. Fernández de Mera, J. de la Fuente);; Universidad Complutense de Madrid, Madrid, Spain (I.G. Fernández de Mera);; Estación Experimental Agropecuaria Rafaela, Santa Fe, Argentina (A.J. Mangold);; Istituto di Patologia Infettiva e Virologia dell'Università di Palermo, Palermo (F. Scarlata);; Oklahoma State University, Stillwater, Oklahoma, USA (J. de la Fuente)

**Keywords:** tick, rickettsia, zoonosis, epidemiology, Rickettsia conorii, Rickettsia slovaca, Mediterranean spotted fever (Boutonneuse fever), Indian tick typhus strain, vector-borne infections, Sicily

**To the Editor:** Rickettsiae are vector-borne pathogens that affect humans and animals worldwide (*1*). Pathogens in the *Rickettsia conorii* complex are known to cause Mediterranean spotted fever (MSF) (*R. conorii* Malish strain), Astrakhan fever (*R. conorii* Astrakhan strain), Israeli spotted fever (*R. conorii* Israeli spotted fever strain), and Indian tick typhus (*R. conorii* Indian tick typhus strain) in the Mediterranean basin and Africa, southern Russia, the Middle East, and India and Pakistan, respectively (*2*). These rickettsioses share some clinical features, such as febrile illness and generalized cutaneous rash, and are transmitted to humans by *Rhipicephalus* spp. ticks (*2*).

MSF is endemic to Sicily (Italy); fatal cases occur each year, and the prevalence of *R. conorii* in dogs is high (*3**–**6*). Recently, *R. conorii* Malish strain and *R. conorii* Israeli spotted fever strain were confirmed in humans in Sicily in whom MSF was diagnosed (*4*), which suggests that other *R. conorii* strains might be present and diagnosed as causing MSF. The rickettsiae within the *R. conorii* complex, which are relevant for the study of bacterial evolution and epidemiology, can be properly identified only by appropriate genetic analyses.

We analyzed 15 blood and 19 inoculation eschar samples collected during 2005–2009 from 31 patients in Palermo Province and 2 in Catania Province, none of whom had recently traveled. None were severely ill, but all 33 had clinical manifestations and laboratory results compatible with MSF: 1-week incubation after tick bite, fever, headache, myalgia, papulonodular rash that started on the upper limbs and spread centripetally with or without tache noire, and detection of antibody titers >180 to *R. conorii* by indirect immunofluorescence antibody test (bioMérieux, Marcy L’Etoile, France).

Total DNA was extracted by using the GeneElute Mammalian Genomic DNA Miniprep Kit (Sigma-Aldrich, Milan, Italy) and used to analyze *Rickettsia* spp. sequences by PCR, cloning, and sequence analysis of the amplicons. At least 3 clones were sequenced for each amplicon. Genes targeted by PCR included ATP synthase α subunit (*atpA*) (*7*), heat-shock protein 70 (*dnaK*) (*7*), outer membrane protein A (*ompA*) (primers Rr190.70p and 190–701 [*8*]), outer membrane protein B (*ompB*) (primers rompBSFGIF and rompBSFG/TGIR [*9*]), citrate synthase (*gltA*) (*2*), and 17-kDa protein (primers TZ15–19 and TZ16–20 [*6*]). Nucleotide sequence identity to reference strains (*2*), multilocus analysis by *atpA*–*dnaK*–*ompA*–*ompB*–*gltA*–*17-kDa* and *ompA*–*ompB* sequences and in silico *Pst*I-*Rsa*I restriction analysis of *ompA* sequences (*8*) were used to characterize *Rickettsia* spp. and *R. conorii* strains.

Results for 15 (45%) patients were positive for *Rickettsia* spp. Thirteen isolates were confirmed as *R. conorii* Malish strain (identification [ID] nos. 44, 45, 47, 49, 54, 55, 57, 59, 61, 66, 68, 92, 112) and 1 each as *R. conorii* Indian tick typhus strain (ID no. 58) and *R. slovaca* (ID no. 50). *R. slovaca* DNA was also found in a *Dermacentor marginatus* tick removed from the patient who had confirmed *R. slovaca* infection. *R. conorii* Malish strains showed 99.9%–100%, 100%, 100%, 98.7%–100%, 100%, and 97.8%–100% pairwise nt sequence identity to reference strain Malish 7 (AE006914) *atpA*, *dnaK*, *ompA*, *ompB*, *gltA*, and 17-kDa protein, respectively.

The *R. conorii* Indian tick typhus strain showed 100%, 100%, 99.4%, 100%, 100%, and 99.9% pairwise nt sequence identity to *R. conorii* strain Malish 7 (AE006914) *atpA*, *dnaK*, 17-kDa protein, and *R. conorii* Indian tick typhus reference strain *ompA* (U43794), *ompB* (AF123726), and *gltA* (U59730), respectively. The *R. slovaca* strain showed 99.4%, 97.8%, 100%, 93.7%, 99.7%, and 99.4% pairwise nt sequence identity to *R. slovaca* *atpA* (AY124734), *dnaK* (DQ821824), *ompA* (HM149286), *ompB* (HQ232242), *gltA* (AY129301), and *R. conorii* strain Malish 7 (AE006914) 17-kDa protein, respectively. The sequences were deposited in GeneBank under accession nos. JN182782–JN182804.

Multilocus sequence analysis (Figure, panel A) and in silico *Pst*I-*Rsa*I restriction analysis of *ompA* sequences also confirmed the identity of the *Rickettsia* spp. we identified. As shown (*2*), multilocus analysis with *ompA*–*ompB* sequences was highly informative about the phylogenetic relationship between *Rickettsia* spp. and *R. conorii* strains (Figure, panel B).

**Figure:**
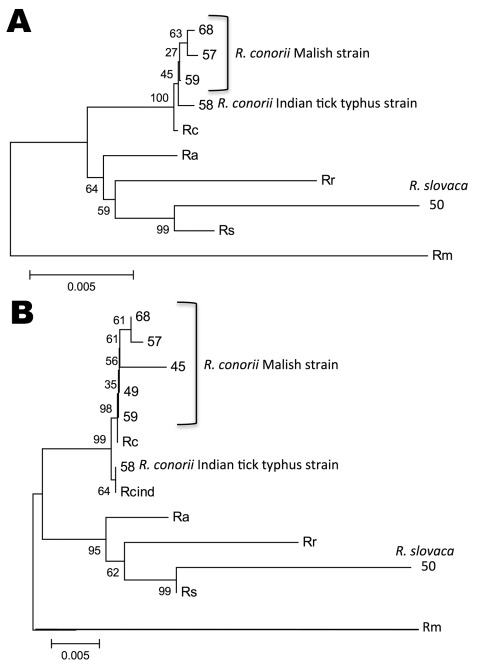
Multilocus sequence analysis of *Rickettsia* spp. Evolutionary history was inferred by using the neighbor-joining method for ATP synthase α subunit (*atpA*)–heat shock protein 70 (*dnaK*)–outer membrane protein A (*ompA*)–*ompB*–citrate synthase (*gltA*)–17-kDa (A) and *ompA*–o*mpB* sequences (B). The optimal tree with the sum of branch length = 0.06205323 (A) and 0.11097561 (B) is shown. The percentage of replicate trees in which the associated taxa clustered together in the bootstrap test (1,000 replicates) are shown next to the branches. The tree is drawn to scale, with branch lengths in the same units as those of the evolutionary distances used to infer the phylogenetic relationship. Evolutionary distances were computed by using the Kimura 2-parameter method and are in the units of the number of base substitutions per site. All ambiguous positions were removed for each sequence pair. Evolutionary analyses were conducted in MEGA5 (www.megasoftware.net). Identification numbers of strains detected are shown with the species/strain name next to them. Rc, *R. conorii* strain Malish 7; Ra, *R. africae* strain ESF-5; Rr, *R. rickettsii* strain Iowa; Rs, *R. slovaca*; Rm, *R. massiliae* strain MTU5; Rcind, *R. conorii* Indian tick typhus strain.

In Sicily, *R. conorii* Malish strain has been characterized in MSF patients (*4*), and *R. slovaca* DNA was identified in ixodid ticks (*5*). However, to our knowledge, *R. slovaca* in humans in Sicily and *R. conorii* Indian tick typhus strain infection in Sicily and Europe have not been reported. The only previous report outside India and Pakistan was documented in a traveler with severe clinical manifestations in France (*10*). Differences were not observed between *R. conorii* Indian tick typhus strain and *R. slovaca*–infected patients. Both patients had similar clinical symptoms compatible with MSF; in both, only IgM for rickettsiae was detected at hospital admission, but IgM and IgG were detected during convalescence. Tache noire were detected in the neck and right arm of patients with *R. conorii* Indian tick typhus strain and *R. slovaca*, respectively.

These results demonstrated that new rickettsiae, such as *R. conorii* Indian tick typhus strain, of public health relevance are emerging in Europe. The widespread distribution of tick vectors in Europe and the transtadial and transovarial transmission of the pathogen in ticks might favor transmission to humans.
